# Predicting how pollinator behavior causes reproductive isolation

**DOI:** 10.1002/ece3.8847

**Published:** 2022-04-19

**Authors:** Robin Hopkins

**Affiliations:** ^1^ The Department of Organismic and Evolutionary Biology and The Arnold Arboretum Harvard University Boston Massachusetts USA

**Keywords:** constancy, pollinator behavior, preference, reproductive isolation, speciation

## Abstract

Pollinator behavior is an important contributor to plants speciation, yet how variation in pollinator behavior causes variation in reproductive isolation (RI) is largely uncharacterized. Here I present a model that predicts how two aspects of pollinator behavior, constancy and preference, contribute to a barrier to reproduction in plants. This model is motivated by two observations: most co‐occurring plants vary in frequency over space and time, and most plants have multiple pollinators that differ in behavior. Thus, my goal was to understand how relative frequencies of plants and pollinators in a community influence ethological RI between co‐occurring plants. I find that RI for a focal plant generally increases with increasing relative plant frequency, but the shape of this relationship is highly dependent on the strength of pollinator behavior (constancy and preference). Additionally, when multiple pollinators express different behavior, I find that pollinators with stronger preference disproportionately influence RI. But, I show that RI caused by constancy is the average RI predicted from constancy of each pollinator weighted by pollinator frequency. I apply this model to examples of pollinator‐mediated RI in *Phlox* and in *Ipomopsis* to predict the relationships between plant frequency and ethological RI in natural systems. This model provides new insights into how and why pollinator specialization causes RI, and how RI could change with changing biological communities.

## Graphical abstract

I create a model that predicts plant ethological reproductive isolation (RI) based on pollinator behavior. This model is motivated by the empirical observation that the relative frequency of hybridizing plants and their pollinators change over space and time. My model predicts how this variation in plant‐pollinator community affects RI between co‐occurring species.

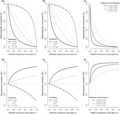

## 1 INTRODUCTION

Animals are necessary vectors for pollen movement between individuals of many flowering plant species (Ollerton et al., 2011). Elaborate floral traits, including variable size, color, shape, and scent have evolved in angiosperms to attract and influence the behavior of pollinators (Campbell et al., 1997; Galen, 1989; Schemske & Bradshaw, 1999; Stanton et al., 1986). Because of the intimate tie between pollinator behavior and reproduction, ethological isolation can be an important barrier to heterospecific mating (reviewed in Grant, 1949; Kay & Sargent, 2009; Van Der Niet et al., 2014). Heterospecific pollen movement can result in hybridization, wasted pollen and, in some cases, stigma clogging (Morales & Traveset, 2008). Thus, from a plant's perspective, pollinator visits are most fruitful if the pollinator moves pollen between conspecific plants.

Speciation involves the evolution of reproductive isolating barriers between diverging lineages. Although there are many components to reproductive isolation (RI) in plants, pollinator behavior can be one of the strongest and most important (reviewed in Baack et al., 2015; Lowry et al., 2008). In many systems, pollinator behavior, as measured in the field, is strongly implicated in the process of plant speciation (e.g., Hopkins & Rausher, 2012; Kay & Schemske, 2003; Klahre et al., 2011; Ramsey et al., 2003; Schmid et al., 2016). Yet, there is still doubt as to the effectiveness of pollinator behavior in inhibiting reproduction and effectively driving speciation (Chittka et al., 1999; Waser, 1998). How can pollinators cause RI when most plants are generalists appealing to a variety of pollinators, and most pollinators are generalists visiting a variety of plants (Jordano, 1987; Ollerton, 2016; Robertson, 1928; Waser et al., 1996)? In order to better address this question, we need a framework for evaluating how quantifiable properties of pollinator behavior can contribute to RI in plants across complex communities.

Although pollinator‐mediated RI between plants can result through both mechanical and behavioral mechanisms (Kay & Sargent, 2009), I focus here on two aspects of pollinator behavior—preference and constancy—that can influence the amount of heterospecific pollen deposition and thus act as a barrier to reproduction. Pollinator preference is the tendency of a pollinator to visit one species or variety of plant more than is expected based on that plant's relative frequency in a population. Preference can be expressed in response to an assortment of traits, such as color, size, shape, and smell, which often act as signals for a reward such as nectar (Schiestl & Johnson, 2013). Preference can cause RI, because in a community with two plant species, a pollinator that strongly prefers one species will transfer pollen between species of plants less than a pollinator that visits both species equally (e.g., Bradshaw & Schemske, 2003; Fulton & Hodges., 1999; Hoballah et al., 2007; Martin et al., 2008).

Pollinator constancy is the tendency of pollinators to move between the same species or variety of plant more than between different plants given what is expected based on the proportion of each plant visited (Waser, 1986). Constancy describes the order of visits to plants rather than the number of visits to each type of plant. High constancy can cause RI when pollen is transferred between conspecific individuals more than between heterospecific individuals. Foraging with constancy has been studied extensively in honeybees and bumblebees (Gegear & Laverty, 2005; Hill et al., 1997; Kephart & Theiss, 2004; Marques et al., 2007; Raine & Chittka, 2005) but has also been documented in Lepidoptera (Aldridge & Campbell, 2007; Goulson & Cory, 1993; Goulson et al., 1997; Hopkins & Rausher, 2012; Kulkarni, 1999; Lewis, 1986) and hoverflies (Goulson & Wright, 1998).

Both constancy and preference are frequently measured in the context of plant speciation and both behaviors have been shown to cause strong RI (Campbell et al., 1997; Fulton & Hodges., 1999; Hopkins & Rausher, 2012; Kay & Sargent, 2009; Kephart & Theiss, 2004; Marques et al., 2007; Schemske & Bradshaw, 1999; Schmid et al., 2016). Most of these studies characterize patterns of pollinator behavior in a small number of locations, with controlled arrays of plants. Although this work strongly supports the important role of pollinators in plant RI, it is not currently possible to infer the strength of RI in natural populations that vary in the relative frequency of plant types and pollinator types. Furthermore, the relative importance of constancy and preference to plant RI has been debated in the literature (Kay & Sargent, 2009; Waser, 1998) but a direct comparison of their respective contributions has not previously been possible.

Co‐occurring plant species vary extensively in relative frequency over space and time. Intuitively, the amount of pollinator movement between species is correlated with the relative frequency of plant species. If a population is predominately made up of one species, even with a pollinator moving randomly between plants, there will be fewer opportunities for transfer of pollen from the rare species to the common species than between the common species. But, we lack an understanding of how aspects of pollinator behavior, such as constancy and preference, affect RI across plant frequencies, especially given what is expected by random movement patterns.

Most of the flowering plants are visited by multiple pollinators (Robertson, 1928; Waser et al., 1996) that do not show the same behavioral responses to floral signals. How is ethological RI affected if one pollinator has strong preference and another has weak preference? What if the frequency of these two pollinators varies across populations? A quantitative framework that describes how multiple pollinators, that express different behaviors, interact to cause RI in plants can provide a more comprehensive understanding of how RI may vary across populations.

Here, I construct a mathematical model that shows how aspects of pollinator behavior contribute to ethological RI. My specific goals are: (1) To define a mathematical relationship between pollinator behavior (constancy and preference) and plant RI, (2) To determine how variation in plant relative frequency affects ethological RI, and (3) To determine how variation in pollinator relative frequency affects pollinator driven RI in a community with multiple pollinators. To demonstrate the relevance of this work to empirical investigations of plant–pollinator interactions, I apply this model to natural examples and predict ethological isolation given preference and constancy observed in the field. With these examples, I demonstrate how standard observations of pollinator movement can be used to predict pollinator mediated RI in the field.

## 2 METHODS

I present a deterministic model describing how pollinator behavior (constancy and preference) can lead to pollinator‐mediated RI across plant and pollinator relative frequencies. The notation used in my model is defined in Table 1. I will first describe a system with a single pollinator and two plants in which one plant is the focal species and the other is the heterospecific pollen donor. Then, I will describe a system with two pollinators and two plant species. Appendix A describes a generalized model that allows for unlimited plants and pollinators in a community. Like most studies of RI, I focus here on a focal plant.

**TABLE 1:** Summary of notation and definitions for model

Notation	Description
${\mathrm{RI}}_{i}$	Strength of Reproductive Isolation due to pollinator *i* behavior varying heterospecific pollen deposition
$H_{i}$	Proportion of heterospecific movements to focal plant compared to total heterospecific and conspecific movements by pollinator *i*
$\rho_{i}$	Pollinator preference of pollinator *i* for focal plant
$\kappa_{i}$	Pollinator constancy of pollinator *i* for focal plant
f	Relative frequency of focal plant in plant community
$\psi_{i}$	Proportion of a pollinator *i* visits made to a focal plant compared to other plants in the community
$v_{i}$	Proportion of focal plant's total visits made by pollinator *i* compared to visits by all other pollinators together
$\phi_{i}$	Proportion of visits to any plant in the community made by pollinator *i* compared to total visits by all other pollinators across the community

### 2.1 One pollinator model

For a given pollinator *i*, the proportion of visits to a focal plant species ($\psi_{i}$) can be described as a function of pollinator preference ($\rho_{i}$) for the plant and the relative frequency of the focal plant (*f*).

(1) $$\psi_{i}=\frac{f(1+\rho_{i})}{1+\rho_{i}\left(2f‐1\right)}$$

Equation (1) is modified from a foraging preference function used by Smithson and MacNair (1996, 1997). Here, preference varies from −1 (never visits focal plant) to 1 (only visits focal plant) with *ρ* = 0 indicating no preference such that the proportion of visits is equal to the frequency of the focal plant. This modification makes preference on the same scale as constancy. Preference for a given plant type is always relative to at least one other plant type in the community. This preference function can be adapted to include frequency dependent variation in preference (see Appendix B).

Pollinator constancy ($\kappa_{i}$) describes how pollinators move between plants given the number of each plant type visited. Constancy can be calculated from the observed proportion of heterospecific movements ($H_{i}$) made by pollinator *i* to a plant and the observed proportion of visits to the focal plant species by the pollinator ($\psi_{i}$).

(2) $$\kappa_{i}=\frac{\left(1‐H_{i}\right)‐\psi_{i}}{\left(1‐H_{i}\right)+\psi_{i}‐2\psi_{i}\left(1‐H_{i}\right)}$$

Equation (2) is modified from the constancy formula presented in Gegear and Thomson (2004). I have modified this equation to calculate constancy to just a focal species instead of total constancy in a community including both species. Constancy varies between −1 and 1, with negative constancy indicating more heterospecific transitions than expected and positive constancy indicating more conspecific transitions than expected. Equation (2) can be solved for *H* in terms of constancy and then be expanded to create an equation describing the proportion of heterospecific movements, or transitions, by pollinator *i* in terms of preference, constancy, and frequency of plants.

(3) $$H_{i}=‐\frac{(f‐1)(\rho_{i}‐1)\left(\kappa_{i}‐1\right)}{1+\rho_{i}\left(2f‐1\right)+\kappa_{i}(2f‐1)+\kappa_{i}\rho_{i}}$$

RI is defined as the reduction in heterospecific gene flow caused by a particular trait. I am interested in the reduction of heterospecific pollen movement caused by behavior (preference and constancy) and thus need to also calculate the expected heterospecific movement with no constancy and preference given plant frequency. In equation (3), if $\rho_{i}$ and $\kappa_{i}$ are zero, then $H_{i}=\left(1‐f\right)$. To be consistent with standard measures of RI, as justified by Sobel and Chen, I define RI to vary from −1 (disassortative mating) to 1 (complete assortative mating) (Sobel & Chen, 2014). Thus, RI caused by pollinator *i* preference and constancy can be defined as:

(4a) $${\mathrm{RI}}_{i}=1+2\left(\frac{H_{i}}{H_{i}+\left(1‐f\right)}\right)$$

where (1 − *f*) represents the expected heterospecific movement for a given plant frequency with no pollinator preference and constancy. Expanded, this simplifies to the following equation for RI:

(4b) $${\mathrm{RI}}_{i}=1+2\left(\frac{f(\rho_{i}+\kappa_{i})}{1+\rho_{i}\left(f‐1\right)+\kappa_{i}(f‐1)+\kappa_{i}\rho_{i}}\right)$$

Using equation (4b), I determine how ethological RI varies across a focal plant's relative frequencies for no, strong, and weak pollinator preference and constancy. It is worth noting that based on this equation, preference and constancy will have equivalent effects on ethological RI.

### 2.2 Two pollinator model

Most plants receive visits from multiple pollinators that have different strength preference and constancy. The total ethological RI for a plant is determined by the heterospecific movements by each pollinator proportional to each pollinator's contribution to the focal plant's visits (shown here for two pollinators and generalized for any number of pollinators in Appendix A).

(5) $$H_{\mathrm{Tot}}=\upsilon_{1}H_{1}+\left(1‐\upsilon_{1}\right)H_{2}$$

where $H_{T}$ is the total proportion of heterospecific pollinator visits to a focal plant. $H_{1}$ and $H_{2}$ are the proportion of heterospecific visits by each of pollinator 1 and pollinator 2, respectively, and $v_{1}$ is the proportion of a focal plant's total visits made by pollinator 1. I can define $v_{1}$ as

(6) $$v_{1}=\frac{\phi_{1}\psi_{1}}{\phi_{1}\psi_{1}+{(1‐\phi }_{1}{)\psi }_{2}}$$

where $\phi_{1}$ is the proportion of visits pollinator 1 makes across all plants in the community out of the total visits by both the pollinators in the community. This is an observed value in the field that reflects not just the relative frequency of a pollinator in a population but also the number of visits a pollinator tends to make relative to other pollinators in the community. Additionally, this is distinct from $\psi_{1}$ (and $\psi_{2}$), which is the proportion of pollinator 1's visits (or pollinator 2's visits) that are to just the focal plant and not to the heterospecific pollen donor plant. As above, $\psi_{1}$ and $\psi_{2}$ are functions of pollinator preference and plant frequency (equation 1). The total RI (${\mathrm{RI}}_{\mathrm{Tot}}$) caused by pollinator movement to a focal species is reflective of the total proportion of heterospecific pollinator visits ($H_{\mathrm{Tot}}$) and the expected number of heterospecific movements given plant frequency (1 − *f*).

(7) $${\mathrm{RI}}_{\mathrm{Tot}}=1‐2\left(\frac{H_{\mathrm{Tot}}}{H_{\mathrm{Tot}}+\left(1‐f\right)}\right)$$

By expanding $H_{\mathrm{Tot}}$, the total ethological RI can be predicted from pollinator behavior (preference and constancy), plant relative frequency, and the proportion of visits by pollinator 1 across the plant community. Expansion of Equation (7) is unwieldy and not easily simplified. The code for this model and instructions for running the model with field‐collected pollinator observation data are found in Appendices A and C as well as here: https://github.com/PhloxHopkins/Pollinator_RI_model.

Using equation (7), I first determine how pollinator‐mediated RI varies across focal plant relative frequency, when multiple pollinators have strong, weak, or no preference and constancy. Second, I determine how pollinator‐mediated RI varies across proportion of pollinator visits when different pollinators have strong, weak, or no preference.

### 2.3 Model applications

I apply this model to empirical datasets of pollinator behavior. Details of how to identify and calculate the necessary parameters from field observations are described in Appendix C.

#### 2.3.1 Pollinator preference and constancy for *Phlox*

Flower color divergence in *P*. *drummondii* is due to reinforcement (Hopkins & Rausher, 2012). A change in flower color, from light‐blue to dark‐red, evolved in sympatric populations in response to selection to increases RI between *Phlox drummondii* and *P*. *cuspidata* (Hopkins & Rausher, 2012). Specifically, *Battus philenor* (pipevine swallowtail) has significantly lower constancy when both the *Phlox* have the ancestral light‐blue flower color then when *P*. *drummondii* has dark‐red flower color (Hopkins & Rausher, 2012). Observations were performed when the focal *P*. *drummondii* plants had a relative frequency of 0.25. How does RI for light‐blue and dark‐red plants vary across different frequencies of *Phlox*, as you might expect to find in the wild? Based on the field observations of *B*. *philenor* (as reported in table S6 of Hopkins & Rausher, 2012) I used equations (1) and (2) to calculate preference and constancy for *B*. *philenor*, and equations (4a) and (4b) to calculate heterospecific movement and ethological RI across *Phlox* frequencies.

#### 2.3.2 Two pollinators' preferences for *Ipomopsis*

*Ipomopsis aggregata* and *Ipomopsis tenuituba* is a classically studied species pair for which pollinator behavior is known to play an important role in RI (Aldridge & Campbell, 2007; Grant & Grant, 1965). These two species differ in several floral traits that influence pollinator preference such that hummingbirds prefer *I*. *aggregata* and hawkmoths prefer *I*. *tenuituba* (Aldridge & Campbell, 2007; Campbell, 2004). Based on the pollinator observation data reported in Table 1 of Aldridge and Campbell (2007), I calculate pollinator preference and constancy for each species at two natural locations of the *Ipomopsis* species and use equation (7) to estimate RI across plant relative frequencies and across proportion of pollinator visits.

## 3 RESULTS

### 3.1 One pollinator model

**FIGURE 1:**
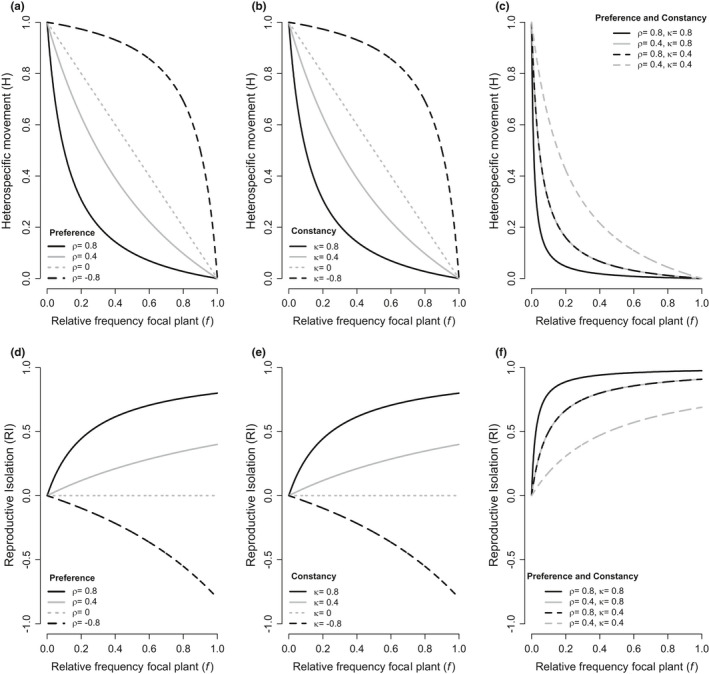
The predicted heterospecific movement (a–c) and reproductive isolation (RI) (d–f) caused by pollinator preference and constancy across plant relative frequencies. Examples in (a and d) for pollinator with no constancy (*κ* = 0) and strong preference for a plant (*ρ* = 0.8, solid black line), weak preference for a plant (*ρ* = 0.4, solid gray line), no preference for plant (*ρ* = 0, dotted gray line), and strong preference against a plant (*ρ* = −0.8, dashed black line) show how the H function is convex, linear, or concave depending on the strength and direction of preference, while the sign of the RI function and the steepness of the curve depends on the strength and direction of preference. Examples (b and e) for pollinators with no preference (*ρ* = 0) and strong constancy (*κ* = 0.8, black solid line), weak constancy (*κ* = 0.4, gray solid line), no constancy (*κ* = 0, dotted gray line), and negative constancy (*κ* = −0.8, dashed black line). In (c and f), examples of pollinators with strong preference and strong constancy (solid black line), weak preference and strong constancy (dashed black line), strong preference and weak constancy (solid gray line), and weak preference and weak constancy (dashed gray line) show that constancy and preference have the same effect on RI

In general, the strength of ethological RI increases with the strength of pollinator preference favoring a focal species (Figure 1). With no preference, the proportion of heterospecific pollinator movements (H) is proportional to the relative frequency of focal plant and RI is zero (Figure 1a,d). When a pollinator has preference for the focal species, H is a decreasing concave function of relative plant frequency and RI is an increasing concave function of relative plant frequency, with the steepness of both curves determined by the strength of preference. When preference is strong (ρ=0.8) and the focal plant is rare, small changes in plant frequency can result in large increases in RI. When preference is weak (ρ=0.4), RI increases less with an equivalent change in plant relative frequency. Intuitively, this means that when a pollinator strongly favors a particular focal species it will continue to visit and transition between individuals of that focal species even as the plant becomes rare. When a pollinator has preference against the focal species, H is a decreasing convex function of relative plant frequency causing RI to be a decreasing concave function of focal plant frequency. RI is always negative with preference against the focal species.

As is evident in equation (4b), variation in constancy effects ethological RI in the same way as preference (Figure 1b,e). With positive constancy, RI increases with plant frequency, and with negative constancy RI decreases with frequency (Figure 1e). Because of the equivalency of these two behaviors toward RI, a pollinator with strong preference and weak constancy causes the same proportion of heterospecific matings and RI as a pollinator with strong constancy and weak preference (Figure 1c,f).

### 3.2 Two pollinator model

**FIGURE 2:**
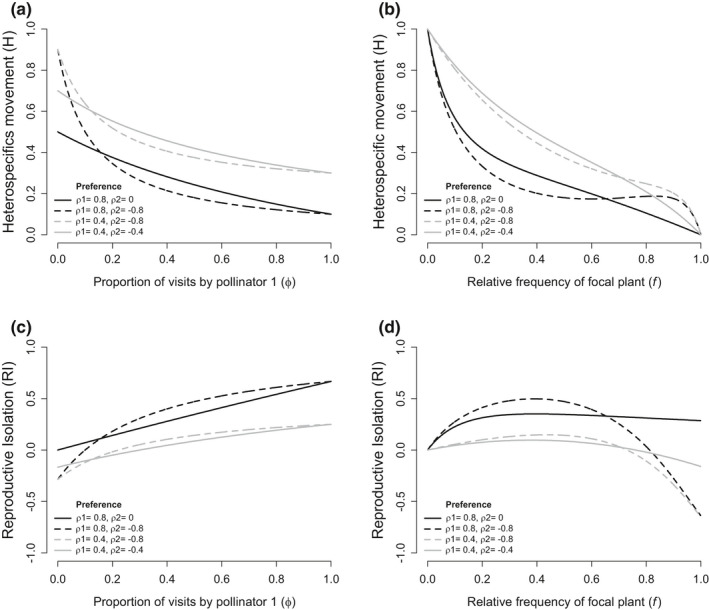
The predicted heterospecific movement (H) and ethological reproductive isolation (RI) when two pollinators differ in preference across proportion of pollinators visits (a and c), and plant relative frequency (b and d). Plots show examples of pollinators with no constancy (*κ* = 0), but different strengths of preference. Black lines are scenarios with one pollinator having strong preference (*ρ*_1_ = 0.8), and gray lines are when one pollinator has weak preference (*ρ*_1_ = 0.4). Solid lines show when a second pollinator has no preference (*ρ*_2_ = 0, black) or weak preference against focal plant (*ρ*_2_ = −0.4, gray) and dashed lines show when a second pollinator has strong preference against the focal plant (*ρ*_2_ = −0.8). Note how dashed RI lines rise above solid lines across most pollinator frequencies (c and d)

By adding a second pollinator to the model, I show how different strengths of preference and constancy interact to predict heterospecific movement of pollen and cause total ethological RI for a focal plant in a community of pollinators. First, I display how multiple pollinators that differ in preference affect predicted heterospecific movement between plants (Figure 2a) and ethological RI (Figure 2c). If plant relative frequency is held at 0.5, H and thus RI are nonlinear functions of the proportion of visits by a pollinator in the system (Figure 2a,c). This reveals that the predicted heterospecific movement from two pollinators with different preference is not simply the average H caused by preference of each pollinator, as would be indicated by a straight line. Instead, the pollinator with stronger preference for the focal plant disproportionally influences the proportion of heterospecific matings and the strength of RI. The magnitude of this deviation from the linear average is proportional to the difference in preference between the two pollinators. The model demonstrates that a plant has stronger RI when it has a pollinator that has strong preference against it, than if it has a pollinator with no preference at all (i.e., the black solid line is below the black dashed line in Figure 2c) across most pollinator frequencies. This is because a pollinator with preference against the focal plant will actively avoid it and therefore represent very few of the visits to the focal plant, but a pollinator with no preference will move freely between species and represent a more significant proportion of visits to the focal plant.

Second, I evaluate how H and RI vary across plant relative frequency when two pollinators are at equal frequency and differ in their preference (Figure 2b,d). The function predicting heterospecific movement is nonlinear with large changes in H occurring due to small changes in frequency when frequencies are extreme. Under some conditions, H becomes a non‐monotonic function of plant frequency such that H decreases as focal plant frequency increases. This dynamic results in RI increasing as a rare focal plant increases in frequency, but as a focal plant becomes frequent, RI decreases and can even become negative at high frequencies. Of note, across many plant frequencies (i.e., 0 < *f* < 0.67 in Figure 2d, dotted line is above solid line) a focal plant has greater RI if pollinator 2 has strong preference against the focal plant than if pollinator 2 has no preference at all.

**FIGURE 3:**
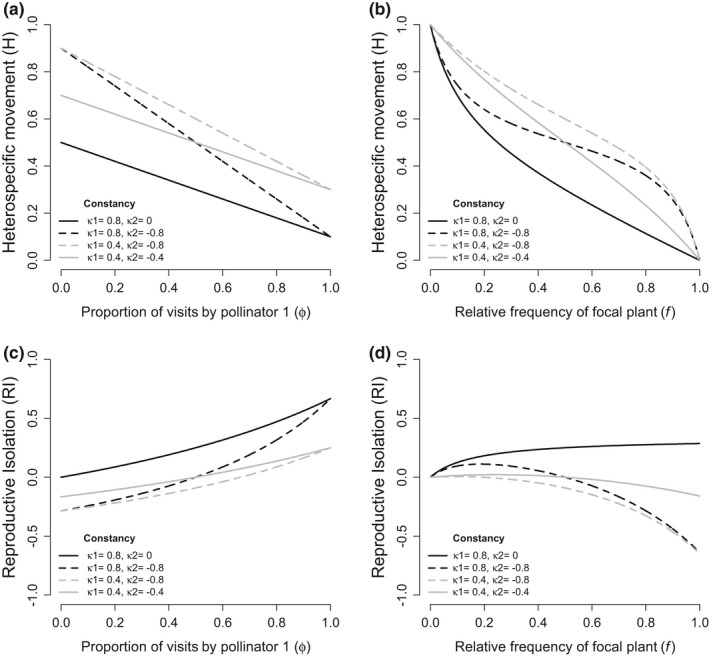
The strength of heterospecific movement (a and b) and ethological reproductive isolation (c and d) when two pollinators differ in constancy across proportion of pollinator visits (a and c), and plant relative frequency (b and d). Plots show examples of pollinators with no preference (*ρ* = 0.0), but different strengths of constancy. Black lines are scenarios with one pollinator having strong constancy (*κ*_1_ = 0.8), and gray lines are when one pollinator has weak constancy (*κ*_1_ = 0.4). Solid lines show when a second pollinator has no constancy (*κ*_2_ = 0, black) or weak negative constancy (*κ*_2_ = −0.4, gray) and dashed lines show when a second pollinator has strong negative constancy (*κ*_2_ = −0.8)

Finally, I show how H and RI vary across proportion of pollinator visits (Figure 3a,c) and relative frequency of focal plant (Figure 3b,d) when pollinators have no preference but vary in constancy. In a two‐pollinator system, the total heterospecific transitions is the average H predicted by each pollinator independently. There is a linear relationship between H and proportion of pollinator visits from pollinator 1 and the slope of the line is determined by the difference in the strength of constancy between the two pollinators. RI is a convex function of proportion of pollinators. Note, unlike with preference, a plant always has greater RI when one of its pollinators has no constancy than when a pollinator has negative constancy (black solid line is above the black dashed line, Figure 3c).

### 3.3 Model applications

#### 3.3.1 Pollinator preference and constancy for *Phlox*

**FIGURE 4:**
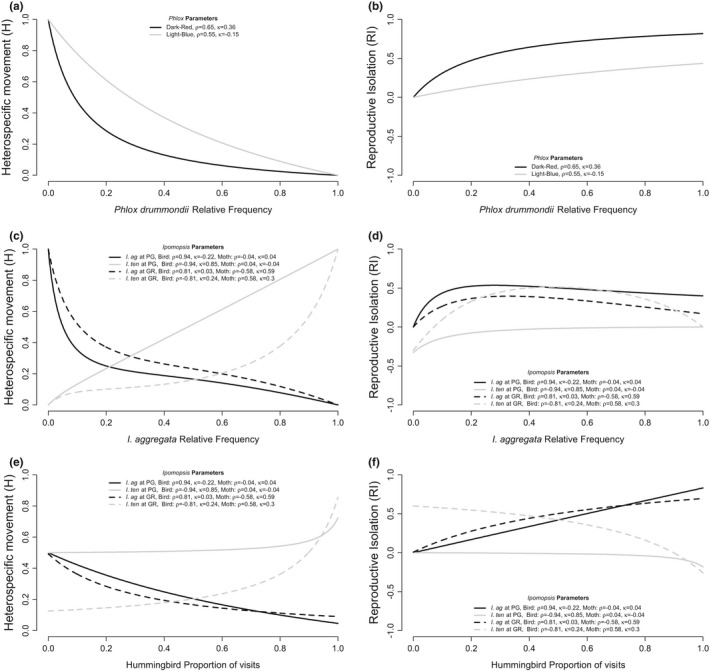
Predicted heterospecific movement and reproductive isolation in natural systems. H (a) and RI (b) of dark‐red and light blue *Phlox drummondii* with *P*. *cuspidata* across *P*. *drummondii* relative frequency. H (c) and RI (d) between *Ipomopsis aggregata* and *Ipomopsis tenuituba* across *I*. *aggregata* relative frequency at two sites (Grizzly Ridge [GR] and Poverty Gulch [PG]) as predicted by behavior of hummingbirds (bird) and hawkmoths (moth). H (e) and RI (f) for each *Ipomopsis* species with equal relative frequency (*f* = 0.5) across proportion of hummingbird visits

I applied the one‐pollinator model to the *Phlox* system using field observations of pollinator constancy and preference for two different *P*. *drummondii* flower color morphs and *P*. *cuspidata*. I found that across all *P*. *drummondii* frequencies, dark‐red flowers are expected to have lower H and higher RI than light‐blue flowers (Figure 4a,b). As noted previously, this difference in ethological RI is due to variation in *B*. *philenor* constancy based on flower color. The difference between RI for two colors decreases when *P*. *drummondii* is rare. Since the selective advantage of the dark‐red color (the strength of reinforcing selection) is determined by the difference between RI of the ancestral light‐blue color and the derived dark‐red color, these results suggest that the strength of reinforcing selection is dependent on the frequency of the two species of plants in the population. At low frequency the dark‐red and light‐blue *P*. *drummondii* are predicted to have similar ethological isolation.

#### 3.3.2 Two pollinators' preferences for *Ipomopsis*

In a second example, I evaluate how H and RI between *Ipomopsis* taxa is predicted to vary across plant frequency given the pollinator preference and constancy observed at two different field sites (PG and GR). Hummingbirds show strong preference for *I*. *aggregata* causing lower heterospecific movement and significant RI with *I*. *tenuituba* at both sites across most relative frequencies of the focal plant (Figure 4c,d, black lines). In one site (PG) preference is stronger than at the other site (GR) causing consistently stronger ethological RI at this site despite variation in hawkmoth behavior. For *I*. *tenuituba* (Figure 4c, gray lines), hawkmoth preference against *I*. *aggregata* and favoring *I*. *tenuituba* causes stronger RI at the GR site then at the PG site where hawkmoths display no preference and RI is negative (Figure 4c, dashed gray line is above solid gray line). The model also predicts how variation in the relative contribution of hummingbird and hawkmoth pollination causes variation in *Ipomopsis* H and RI (Figure 4e,f). Because hummingbirds show strong preference for *I*. *aggregata*, RI for this species steadily increases with increasing frequency of birds. The strong preference against *I*. *tenuituba* means humming bird proportion of visits has very little effect on RI for this species until birds make up nearly all of the visits.

## 4 DISCUSSION

I developed a model for understanding how the frequency of hybridizing plants and the frequency of pollinators affects pollinator mediated ethological RI. This research focuses on two aspects of pollinator behavior contributing to plant speciation—preference and constancy—that describe which plants get visits and the order of plant visitation (Fenster et al., 2004; Kay & Sargent, 2009).

### 4.1 Model implications

My research is motivated by the observation that plant‐pollinator communities vary over geographic space and time and yet we lack a clear understanding of the implications of this variation for plant RI. As discussed below, other authors have addressed this problem in a variety of ways. I took an analytical approach to derive the simplest model based on quantifiable aspects of pollinator behavior, relative frequency of hybridizing plant species, and relative frequency of pollinator visits in the system to predict RI for a plant.

My model reveals some intuitive results—for example, RI increases as pollinator preference and constancy increase—as well as some less‐intuitive results. I found that RI usually increases as focal plant frequency increases in a community, but that the relationship between RI and plant frequency is nonlinear with the shape of this curve determined by the strength of pollinator preference and constancy. The stronger the behavior, the steeper the curve, such that strong preference or constancy leads to high RI even at low focal plant relative frequencies. This means that at edges of hybrid zones, strong pollinator preference may still lead to strong RI for the rare species.

Most plant communities have multiple pollinators, and multiple pollinators make the relationship between plant frequency and RI more complex. When two pollinators differ in their preference, their contributions to total RI is not additive. The pollinator that more strongly prefers the focal species disproportionately contributes to RI. A pollinator that disfavors a focal plant has a negligible impact on total RI until the focal species is very common at which point this pollinator drags down total RI. All else being equal, a focal plant will experience a higher RI if one pollinator prefers the focal plant and a second pollinator has preference against the focal plant than if the second pollinator has no preference at all.

The nonadditive contributions of two pollinators to RI reveals a mechanism for how pollinator specialization is favored. It has long been assumed, and observed, that if two closely related plant species attract different pollinators that express opposing preferences, then this specialization will result in RI between the plants (Fenster et al., 2004; Kay & Sargent, 2009). The advantage of pollinator specialization has been an emergent property in other theoretical models describing plant‐pollinator communities (Sargent & Otto, 2006). If we consider pollinator specialization to occur when one pollinator has strong preference favoring the focal plant and other pollinators in the community disfavors or avoids the plant, then my model demonstrates how pollinator specialization is favored over having one pollinator with preference for the focal plant in a community of pollinators that have no preference or lack specialization. A plant will experience less heterospecific pollen deposition if it discourages visitation by non‐choosy pollinators because of the way in which preference of two pollinators is not additively combined to cause RI. In other words, it may be beneficial to evolve traits that deter a generalist pollinator so as to avoid pollen transfer from heterospecific plants (as has been hypothesized for parapatric *Clarkia* species (Kay et al., 2019)). Unlike with preference, constancy from two different pollinators does combine additively to predict RI.

Despite specialization causing greater RI, most plants and pollinators are generalists. Yet, even for systems in which multiple pollinators visit a focal species and all pollinators visit multiple plants, ethological RI can significantly contribute to maintaining species boundaries (Keller et al., 2021; Ma et al., 2019). Now, with this model, empiricists can incorporate data from multiple pollinators into one estimate of ethological RI and predict how changing pollinator communities might cascade to changes in hybridization.

### 4.2 Model applications

The results from my model provide expectations for how pollinator mediated RI should vary across populations that differ in plant and pollinator composition. A strength of my model is that empirical observations of pollinator behavior in natural or artificial plant communities can be used to predict pollinator mediated RI across a variable landscape. Specifically, using field observations of number and pattern of pollinator visits, this model can predict ethological RI across any pollinator or plant relative frequency that occurs in nature.

This model can be applied to predict how RI varies across a variety of important natural scenarios. For example, hybrid zones often result when two ranges form overlapping reciprocal clines in relative plant frequency. Generally, my model predicts that RI is inversely related to frequency, but depending on the pollinator behavior, both species of plants may have strong RI across most relative plant frequencies (as in the *Ipomopsis* GR site, dashed lines Figure 4b). This model can also make predictions about how hybridization will increase or decrease as climate change shifts plant and pollinator ranges, phenology, and population sizes (Memmott et al., 2007). For example, if a particular pollinator is predicted to decrease in abundance, RI for a plant preferred by that pollinator might actually be fairly stable until the pollinator is nearly extinct (as in Figure 1a).

The predictions from my model are based on simplifying assumptions including: (1) Pollinator preference and constancy are constant, (2) The relevant spatial scale of plant populations and pollinator foraging populations align, (3) There are no spatially dependent aspects of pollinator foraging within the relevant scale of inquiry. It is likely that natural plant‐pollinator communities are more complicated than described by my model and violate some of these assumptions. For example, some pollinators show frequency dependent preference (e.g., Cresswell & Galen, 1991; Gigord et al., 2001; Smithson & MacNair, 1996; Smithson & MacNair, 1997) and, as seen in Appendix A, this changes the shape of the RI function across populations. In other systems pollinators may vary their behavior depending on the behavior and frequency of other pollinators in the community (e.g., Brosi & Briggs, 2013; Fontaine et al., 2008; Inouye, 1978).

The simplified model presented here can act as a null against which variation in natural systems can be tested. Empiricists can observe plant and pollinator frequencies and movement patterns across multiple populations, calculate preference and constancy and use the presented model to estimate ethological RI. If the variation in the calculated RI across the populations does not fit the RI predicted by the model based on observed frequency and movement patterns, then the model can be updated. For example, frequency dependent behavior can be added to the model (Appendix B) or variation in preference due to pollinator or plant assemblage could be tested (as in Natalis & Wesselingh, 2013). In other words, if natural variation in RI does not conform to the expectations of the model, we now have a powerful framework with which to test alternative hypotheses about what other factors affect ethological RI.

My model focuses specifically on ethological RI and does not consider mechanical RI or total fitness of a plant. In other words, the model assumes that there are always enough pollinators to pollinate flowers. I acknowledge that in a pollinator limited system, the actual RI a plant experiences is not the only, or maybe even the most important, source of selection. The model currently does not take into account variation in efficiency of pollen transfer (as in the simulation model in Campbell et al., 2002) and in some systems this assumption is clearly violated (Ashman et al., 2004; Burd, 1994; Larson & Barrett, 2000). Extending the model to incorporate such scenarios is an important future direction and could easily be done by modifying equation (6). Depending on the specifics of a system of interest, an efficiency term that modifies either the proportion of visits to any plant in the community or the proportion of pollinator visits made to a focal plant could be added to the basic model presented here.

Previous theoretical research has considered aspects of plant‐pollinator community context to understand pollinator specialization, pollinator network structure (Bascompte et al., 2006), and how perturbations disrupt pollination (Kaiser‐Bunbury et al., 2010; Memmott et al., 2004; Ramos‐Jiliberto et al., 2012). Models such as these provide important insights into how and why plant‐pollinator communities might be structured as they are and how their current structure can maintain biodiversity and community stability. Some of these models have even incorporated explicit aspects of pollinator behavior, such as adaptive foraging (Valdovinos et al., 2013, 2016). My model adds to this literature by specifically describing the implications of perturbations to pollination networks for speciation and potential hybridization.

## 5 CONCLUSIONS

The goal of my model was to deconstruct the quantifiable aspects of pollinator behavior to understand how variation in behavior contributes to RI across different plant communities. The model can generate predictions about how temporal and spatial fluctuations in pollinator composition and plant composition influences pollinator ethological RI. This can be useful for empiricists to better understand how the pollinator behavior they measure in the field leads to RI across changing communities. To understand plant speciation it is important to determine how components of RI may vary across space and time. Finally, this model is worthwhile at a theoretical level in that it brings together commonly used, yet seemingly unrelated, equations for pollinator behavior and RI to analytically describe ethological isolation in plants.

## AUTHOR CONTRIBUTIONS

**Robin Hopkins:** Conceptualization (lead); Formal analysis (lead); Funding acquisition (lead); Investigation (lead); Methodology (lead); Project administration (equal); Visualization (lead); Writing – original draft (lead); Writing – review & editing (lead).

### OPEN RESEARCH BADGES

This article has earned an Open Data Badge for making publicly available the digitally‐shareable data necessary to reproduce the reported results. The data is available at https://github.com/PhloxHopkins/Pollinator_RI_model.

### DATA AVAILABILITY STATEMENT

All code for generating the model, running the model, and making the figures is available at https://github.com/PhloxHopkins/Pollinator_RI_model.

## Supporting information

Appendix S1‐S3

Appendix S4

## References

[ece38847-bib-0001] Aldridge, G. , & Campbell, D. R. (2007). Variation in pollinator preference between two Ipomopsis contact sites that differ in hybridization rate. Evolution, 61, 99–110. 10.1111/j.1558-5646.2007.00008.x 17300430

[ece38847-bib-0002] Ashman, T. L. , Knight, T. M. , Steets, J. A. , Amarasekare, P. , Burd, M. , Campbell, D. R. , Dudash, M. R. , Johnston, M. O. , Mazer, S. J. , Mitchell, R. J. , Morgan, M. T. , & Wilson, W. G. (2004). Pollen limitation of plant reproduction: Ecological and evolutionary causes and consequences. Ecology, 85, 2408–2421. 10.1890/03-8024

[ece38847-bib-0003] Baack, E. , Melo, M. C. , Rieseberg, L. H. , & Ortiz‐Barrientos, D. (2015). The origins of reproductive isolation in plants. New Phytologist, 207, 968–984. 10.1111/nph.13424 25944305

[ece38847-bib-0004] Bascompte, J. , Jordano, P. , & Olesen, J. (2006). Asymmetric coevolutionary networks facilitate biodiversity maintenance. Science, 312, 431–433. 10.1126/science.1123412 16627742

[ece38847-bib-0005] Bradshaw, H. D. , & Schemske, D. W. (2003). Allele substitution at a flower colour locus produces a pollinator shift in monkeyflowers. Nature, 426, 176–178. 10.1038/nature02106 14614505

[ece38847-bib-0006] Brosi, B. J. , & Briggs, H. M. (2013). Single pollinator species losses reduce floral fidelity and plant reproductive function. Proceedings of the National Academy of Sciences, 110, 13044–13048. 10.1073/pnas.1307438110 PMC3740839 23878216

[ece38847-bib-0007] Burd, M. (1994). Bateman's principle and plant reproductiction: The role of pollin limiation in fruit and seed set. The Botanical Review, 60, 83–139.

[ece38847-bib-0008] Campbell, D. R. (2004). Natural selection in Ipomopsis hybrid zones: Implications for ecological speciation. New Phytologist, 161, 83–90.

[ece38847-bib-0009] Campbell, D. R. , Waser, N. M. , & MelendezAckerman, E. J. (1997). Analyzing pollinator‐mediated selection in a plant hybrid zone: Hummingbird visitation patterns on three spatial scales. American Naturalist, 149, 295–315. 10.1086/285991

[ece38847-bib-0010] Campbell, D. R. , Waser, N. M. , & Pederson, G. T. (2002). Predicting patterns of mating and potential hybridization from pollinator behavior. The American Naturalist, 159, 438–450. 10.1086/339457 18707428

[ece38847-bib-0011] Chittka, L. , Thomson, J. D. , & Waser, N. M. (1999). Flower constancy, insect psychology, and plant evolution. Naturwissenschaften, 86, 361–377. 10.1007/s001140050636

[ece38847-bib-0012] Cresswell, J. E. , & Galen, C. (1991). Frequency‐dependent selection and adaptive surfaces for floral character combinations ‐ The pollination of *Polemonium viscosum* . American Naturalist, 138, 1342–1353. 10.1086/285290

[ece38847-bib-0013] Fenster, C. B. , Armbruster, W. S. , Wilson, P. , Dudash, M. R. , & Thomson, J. D. (2004). Pollination syndromes and floral specialization. Annual Review of Ecology Evolution and Systematics, 35, 375–403. 10.1146/annurev.ecolsys.34.011802.132347

[ece38847-bib-0014] Fontaine, C. , Collin, C. L. , & Dajoz, I. (2008). Generalist foraging of pollinators: Diet expansion at high density. Journal of Ecology, 96, 1002–1010. 10.1111/j.1365-2745.2008.01405.x

[ece38847-bib-0015] Fulton, M. , & Hodges, S. A. (1999). Floral isolation between *Aquilegia formosa* and *Aquilegia pubescens* . Proceedings of the Royal Society B, 266, 2247–2252.

[ece38847-bib-0016] Galen, C. (1989). Measuring pollinator‐mediated selection on morphometric floral traits ‐ Bumblebees and the alpine sky pilot, *Polemondium viscosum* . Evolution, 43, 882–890. 28564200 10.1111/j.1558-5646.1989.tb05185.x

[ece38847-bib-0017] Gegear, R. J. , & Laverty, T. M. (2005). Flower constancy in bumblebees: A test of the trait variability hypothesis. Animal Behaviour, 69, 939–949. 10.1016/j.anbehav.2004.06.029

[ece38847-bib-0018] Gegear, R. J. , & Thomson, J. D. (2004). Does the flower constancy of bumble bees reflect foraging economics? Ethology, 110, 793–805. 10.1111/j.1439-0310.2004.01010.x

[ece38847-bib-0019] Gigord, L. D. B. , Macnair, M. R. , & Smithson, A. (2001). Negative frequency‐dependent selection maintains a dramatic flower color polymorphism in the rewardless orchid *Dactylorhiza sambucina* (L.) Soo. Proceedings of the National Academy of Sciences of the United States of America, 98, 6253–6255. 11353863 10.1073/pnas.111162598 PMC33454

[ece38847-bib-0020] Goulson, D. , & Cory, J. S. (1993). Flower constancy and learning in foraging preferences of the green‐veined white butterfly *Pleris napi* . Ecological Entomology, 18, 315–320. 10.1111/j.1365-2311.1993.tb01107.x

[ece38847-bib-0021] Goulson, D. , Ollerton, J. , & Sluman, C. (1997). Foraging strategies in the small skipper butterfly, *Thymelicus flavus*: When to switch? Animal Behaviour, 53, 1009–1016.

[ece38847-bib-0022] Goulson, D. , & Wright, N. (1998). Flower constancy in the hoverflies *Episyrphus balteatus* (Degeer) and *Syrphus ribesii* (L.)(Syrphidae). Behavioral Ecology, 9, 213–219.

[ece38847-bib-0023] Grant, V. (1949). Pollination systems as isolating mechanisms in angiosperms. Evolution, 3, 82–97. 10.1111/j.1558-5646.1949.tb00007.x 18115119

[ece38847-bib-0024] Grant, V. , & Grant, K. (1965). Flower pollination in the Phlox family. Columbia University Press.

[ece38847-bib-0025] Hill, P. , Wells, P. , & Wells, H. (1997). Spontaneous flower constancy and learning in honey bees as a function of colour. Animal Behaviour, 54, 615–627. 10.1006/anbe.1996.0467 9299046

[ece38847-bib-0026] Hoballah, M. E. , Gubitz, T. , Stuurman, J. , Broger, L. , Barone, M. , Mandel, T. , Dell'Olivo, A. , Arnold, M. , & Kuhlemeier, C. (2007). Single gene‐mediated shift in pollinator attraction in Petunia. The Plant Cell, 19, 779–790. 17337627 10.1105/tpc.106.048694 PMC1867374

[ece38847-bib-0027] Hopkins, R. , & Rausher, M. D. (2012). Pollinator‐mediated selection on flower color allele drives reinforcement. Science, 335, 1090–1092. 10.1126/science.1215198 22300852

[ece38847-bib-0028] Inouye, D. (1978). Resource partitioning in bumblebees: Experimental studies of foraging behavior. Ecology, 59, 672–678. 10.2307/1938769

[ece38847-bib-0029] Jordano, P. (1987). Patterns of mutualistic interactions in pollination and seed dispersal: Connectance, dependence asymmetries, and coevolution. The American Naturalist, 129, 657–677. 10.1086/284665

[ece38847-bib-0030] Kaiser‐Bunbury, C. N. , Muff, S. , Memmott, J. , Müller, C. B. , & Caflisch, A. (2010). The robustness of pollination networks to the loss of species and interactions: A quantitative approach incorporating pollinator behaviour. Ecology Letters, 13, 442–452. 10.1111/j.1461-0248.2009.01437.x 20100244

[ece38847-bib-0031] Kay, K. M. , & Sargent, R. D. (2009). The role of animal pollination in plant speciation: Integrating ecology, geography, and genetics. Annual Review of Ecology Evolution and Systematics, 40, 637–656. 10.1146/annurev.ecolsys.110308.120310

[ece38847-bib-0032] Kay, K. M. , & Schemske, D. W. (2003). Pollinator assemblages and visitation rates for 11 species of neotropical Costus (Costaceae). Biotropica, 35, 198–207.

[ece38847-bib-0033] Kay, K. M. , Zepeda, A. M. , & Raguso, R. A. (2019). Experimental sympatry reveals geographic variation in floral isolation by hawkmoths. Annals of Botany, 123, 405–413. 30032166 10.1093/aob/mcy143 PMC6344223

[ece38847-bib-0034] Keller, B. , Ganz, R. , Mora‐Carrera, E. , Nowak, M. D. , Theodoridis, S. , Koutroumpa, K. , & Conti, E. (2021). Asymmetries of reproductive isolation are reflected in directionalities of hybridization: integrative evidence on the complexity of species boundaries. New Phytologist, 229, 1795–1809. 10.1111/nph.16849 32761901

[ece38847-bib-0035] Kephart, S. , & Theiss, K. (2004). Pollinator‐mediated isolation in sympatric milkweeds (*Asclepias*): Do floral morphology and insect behavior influence species boundaries? New Phytologist, 161, 265–277.

[ece38847-bib-0036] Klahre, U. , Gurba, A. , Hermann, K. , Saxenhofer, M. , Bossolini, E. , Guerin, P. M. , & Kuhlemeier, C. (2011). Pollinator choice in petunia depends on two major genetic loci for floral scent production. Current Biology, 21, 730–739. 10.1016/j.cub.2011.03.059 21497087

[ece38847-bib-0037] Kulkarni, R. N. (1999). Evidence for phenotypic assortative mating for flower colour in periwinkle. Plant Breeding, 118, 561–564. 10.1046/j.1439-0523.1999.00429.x

[ece38847-bib-0038] Larson, B. M. H. , & Barrett, S. C. H. (2000). A comparative analysis of pollen limitation in flowering plants. Biological Journal of the Linnean Society, 69, 503–520. 10.1111/j.1095-8312.2000.tb01221.x

[ece38847-bib-0039] Lewis, A. C. (1986). Memory constraints and flower choice in *Pieris rapae* . Science, 232, 863–865. 17755969 10.1126/science.232.4752.863

[ece38847-bib-0040] Lowry, D. B. , Modliszewski, J. L. , Wright, K. M. , Wu, C. A. , & Willis, J. H. (2008). The strength and genetic basis of reproductive isolating barriers in flowering plants. Philosophical Transactions of the Royal Society B‐Biological Sciences, 363, 3009–3021. 10.1098/rstb.2008.0064 PMC2607309 18579478

[ece38847-bib-0041] Ma, Y. , Marczewski, T. , Xue, D. , Wu, Z. , Liao, R. , Sun, W. , & Marczewski, J. (2019). Conservation implications of asymmetric introgression and reproductive barriers in a rare primrose species. BMC Plant Biology, 19, 286. 10.1186/s12870-019-1881-0 31253088 PMC6599365

[ece38847-bib-0042] Marques, I. , Rossello‐Graell, A. , Draper, D. , & Iriondo, J. M. (2007). Pollination patterns limit hybridization between two sympatric species of Narcissus (*Amaryllidaceae*). American Journal of Botany, 94, 1352–1359. 10.3732/ajb.94.8.1352 21636503

[ece38847-bib-0043] Martin, N. H. , Sapir, Y. , & Arnold, M. L. (2008). The genetic architecture of reproductive isolation in louisiana irises: Pollination syndromes and pollinator preferences. Evolution, 62, 740–752. 10.1111/j.1558-5646.2008.00342.x 18266989

[ece38847-bib-0044] Memmott, J. , Craze, P. G. , Waser, N. M. , & Price, M. V. (2007). Global warming and the disruption of plant‐pollinator interactions. Ecology Letters, 10, 710–717. 10.1111/j.1461-0248.2007.01061.x 17594426

[ece38847-bib-0045] Memmott, J. , Waser, N. M. , & Price, M. V. (2004). Tolerance of pollination networks to species extinctions. Proceedings of the Royal Society of London. Series B: Biological Sciences, 271, 2605–2611. 10.1098/rspb.2004.2909 15615687 PMC1691904

[ece38847-bib-0046] Morales, C. L. , & Traveset, A. (2008). Interspecific pollen transfer: Magnitude, prevalence and consequences for plant fitness. Critical Reviews in Plant Sciences, 27, 221–238. 10.1080/07352680802205631

[ece38847-bib-0047] Natalis, L. C. , & Wesselingh, R. A. (2013). Parental frequencies and spatial configuration shape bumblebee behavior and floral isolation in hybridizing Rhinanthus. Evolution, 67, 1692–1705. 23730762 10.1111/evo.12044

[ece38847-bib-0048] Ollerton, J. (2016). Reconciling ecological processes with phylogenetic patterns: The apparent paradox of plant–pollinator systems. Journal of Ecology, 84, 767–769. 10.2307/2261338

[ece38847-bib-0049] Ollerton, J. , Winfree, R. , & Tarrant, S. (2011). How many flowering plants are pollinated by animals? Oikos, 120, 321–326. 10.1111/j.1600-0706.2010.18644.x

[ece38847-bib-0050] Raine, N. E. , & Chittka, L. (2005). Comparison of flower constancy and foraging performance in three bumblebee species (Hymenoptera: Apidae: Bombus). Entomologia Generalis, 28, 81–89.

[ece38847-bib-0051] Ramos‐Jiliberto, R. , Valdovinos, F. S. , Moisset de Espanés, P. , & Flores, J. D. (2012). Topological plasticity increases robustness of mutualistic networks. Journal of Animal Ecology, 81, 896–904. 10.1111/j.1365-2656.2012.01960.x 22313043

[ece38847-bib-0052] Ramsey, J. , Bradshaw, H. D. , & Schemske, D. W. (2003). Components of reproductive isolation between the monkeyflowers *Mimulus lewisii* and *M. cardinalis* (Phrymaceae). Evolution, 57, 1520–1534. 10.1111/j.0014-3820.2003.tb00360.x 12940357

[ece38847-bib-0053] Robertson, C. (1928). Flowers and insects; lists of visitors of four hundred and fifty‐three flowers. Charles Robertson.

[ece38847-bib-0054] Sargent, R. D. , & Otto, S. P. (2006). The role of local species abundance in the evolution of pollinator attraction in flowering plants. American Naturalist, 167, 67–80. 10.1086/498433 16475100

[ece38847-bib-0055] Schemske, D. W. , & Bradshaw, H. D. (1999). Pollinator preference and the evolution of floral traits in monkeyflowers (Mimulus). Proceedings of the National Academy of Sciences of the United States of America, 96, 11910–11915. 10518550 10.1073/pnas.96.21.11910 PMC18386

[ece38847-bib-0056] Schiestl, F. P. , & Johnson, S. D. (2013). Pollinator‐mediated evolution of floral signals. Trends in Ecology & Evolution, 28, 307–315. 10.1016/j.tree.2013.01.019 23480953

[ece38847-bib-0057] Schmid, B. , Nottebrock, H. , Esler, K. J. , Pagel, J. , Böhning‐Gaese, K. , Schurr, F. M. , Mueller, T. , & Schleuning, M. (2016). A bird pollinator shows positive frequency dependence and constancy of species choice in natural plant communities. Ecology, 97, 3110–3118. 10.1002/ecy.1565 27870050

[ece38847-bib-0058] Smithson, A. , & Macnair, M. R. (1996). Frequency‐dependent selection by pollinators: Mechanisms and consequences with regard to behaviour of bumblebees *Bombus terrestris* (L) (Hymenoptera: Apidae). Journal of Evolutionary Biology, 9, 571–588. 10.1046/j.1420-9101.1996.9050571.x

[ece38847-bib-0059] Smithson, A. , & MacNair, M. R. (1997). Density‐dependent and frequency‐dependent selection by bumblebees *Bombus terrestris* (L) (Hymenoptera: Apidae). Biological Journal of the Linnean Society, 60, 401–417. 10.1111/j.1095-8312.1997.tb01503.x

[ece38847-bib-0060] Sobel, J. M. , & Chen, G. F. (2014). Unification of methods for estimating the strength of reproductive isolation. Evolution, 68(5), 1511–1522. 24450287 10.1111/evo.12362

[ece38847-bib-0061] Stanton, M. L. , Snow, A. A. , & Handel, S. N. (1986). Floral evolution ‐ Attractiveness to pollinators increases male fitness. Science, 232, 1625–1627. 10.1126/science.232.4758.1625 17812141

[ece38847-bib-0062] Valdovinos, F. S. , Brosi, B. J. , Briggs, H. M. , Moisset de Espanés, P. , Ramos‐Jiliberto, R. , & Martinez, N. D. (2016). Niche partitioning due to adaptive foraging reverses effects of nestedness and connectance on pollination network stability. Ecology Letters, 19, 1277–1286. 10.1111/ele.12664 27600659

[ece38847-bib-0063] Valdovinos, F. S. , Moisset de Espanés, P. , Flores, J. D. , & Ramos‐Jiliberto, R. (2013). Adaptive foraging allows the maintenance of biodiversity of pollination networks. Oikos, 122, 907–917. 10.1111/j.1600-0706.2012.20830.x

[ece38847-bib-0064] Van Der Niet, T. , Peakall, R. , & Johnson, S. D. (2014). Pollinator‐driven ecological speciation in plants: New evidence and future perspectives. Annals of Botany, 113, 199–211. 10.1093/aob/mct290 24418954 PMC3890394

[ece38847-bib-0065] Waser, N. M. (1986). Flower constancy ‐ Definition, cause, and measurement. American Naturalist, 127, 593–603. 10.1086/284507

[ece38847-bib-0066] Waser, N. M. (1998). Pollination, angiosperm speciation, and the nature of species boundries. Oikos, 82, 198–201.

[ece38847-bib-0067] Waser, N. M. , Chittka, L. , Price, M. V. , Williams, N. M. , & Ollerton, J. (1996). Generalization in pollination systems, and why it matters. Ecology, 77, 1043–1060. 10.2307/2265575

