# Supplementary material for: Predicting how pollinator behavior causes reproductive isolation
Source: Ecol Evol. 2022 Apr 19;12(4):e8847. doi: 10.1002/ece3.8847 (PMC9019001; doi:10.1002/ece3.8847)
Supplement: Supplementary file 1 — Appendix S1‐S3 [file ECE3-12-e8847-s002.docx]

**Appendix S1: Generalized model for RI from multiple pollinators and multiple plants**

The equations presented in the main text apply to one or two pollinators behaving in reference to two plant species (one of which is the focal species). These equations can be generalized for any number of pollinators.

Equation 5 in main text for total heterospecific movement generalized for *n* pollinators is

$H_{Tot}=\sum_{i=1}^{i=n} \upsilon_{i}H_{i}$ (A1)

where $H_{i}$ is the proportion of heterospecific movements predicted for pollinator *i* as in equation 3 in the main text*,* and $\upsilon_{i}$ is the proportion of total plants visits made by pollinator *i*. Note that $\sum_{i=1}^{i=n} \upsilon_{i}=1$.

Equation 6, in the main text, for the total proportion of visits to a plant made by any pollinator *i* generalized for *n* pollinators is

$v_{i}=\frac{\phi_{i}\psi_{i}}{\sum_{j=1}^{j=n} \phi_{j}\psi_{j}}$ (A2)

where $\phi_{i}$ is the frequency of visits by pollinator *i* across all plants, such that the sum of all $\phi_{i}$ across pollinators 1 through *n* equals 1. For each pollinator, $\psi_{j}$ is the proportion of visits it makes to just the focal plant.

The total ethological reproductive isolation (${RI}_{Total}$) for a give focal plant can be predicted from the heterospecific matings of each of *n* pollinators in a community and the proportion of visits to a plant made by each pollinator.

${RI}_{Total}=1-2*(\sum_{i=1}^{i=n} (H_{i}v_{i}))/(\sum_{i=1}^{i=n} \left( H_{i}v_{i} \right)+(1-f))$ (A3)

Expanding $H_{i}$ and $v_{i}$ gives and unwieldy equation of RI in terms of constancy and preference:

${RI}_{Total}=1-2*\frac{\sum_{i=1}^{i=n} \left( \left( -\frac{\left( f-1 \right)\left( \rho_{i}-1 \right)\left( \kappa_{i}-1 \right)}{1+\rho_{i}\left( 2f-1 \right)+\kappa_{i}(2f-1)+{\kappa_{i}\rho}_{i})} \right)*\frac{\phi_{i}\psi_{i}}{\sum_{j=1}^{j=n} \phi_{j}\psi_{j}} \right)}{\sum_{i=1}^{i=n} \left( \left( -\frac{\left( f-1 \right)\left( \rho_{i}-1 \right)\left( \kappa_{i}-1 \right)}{1+\rho_{i}\left( 2f-1 \right)+\kappa_{i}(2f-1)+{\kappa_{i}\rho}_{i})} \right)*\frac{\phi_{i}\psi_{i}}{\sum_{j=1}^{j=n} \phi_{j}\psi_{j}} \right)+\left( 1-f \right)}$ (A4)

**Appendix S2: Model of Frequency dependent pollinator behavior**

Frequency dependent changes in pollinator preference can occur and will change the predicted proportion of visits by a pollinator to the focal plant ($\psi_{i}$). The below equation describing proportion of visits by a pollinator includes a frequency dependent parameter (*b),* which is the coefficient of frequency-dependence as in Smithson and Macnair (1996):

$\psi_{i}=\frac{\left( \frac{-f\left( 1+\rho_{i} \right)}{\rho_{i}-1} \right)^{b}}{\left( 1-f \right)^{b}+\left( -\frac{f\left( 1+\rho_{i} \right)}{\rho_{i}-1} \right)^{b}}$ (C1)

When *b* =1 there is no frequency dependent change in preference, when *b*>1 preference is stronger for more frequent plants representing positive frequency-dependence, and when *b<*1 preference is less for frequent plants representing negative frequency-dependence.

Pollinator mediated RI for a focal plant is:

${RI}_{i}=1-2(\frac{\frac{\left( \kappa_{i}-1 \right)\left( \psi_{i}-1 \right)}{1-\kappa_{i}+2\kappa_{i}\psi_{i}}}{\frac{\left( \kappa_{i}-1 \right)\left( \psi_{i}-1 \right)}{1-\kappa_{i}+2\kappa_{i}\psi_{i}}+\left( 1-f \right)})$ (C2)

Frequency dependent selection sharpens the curve of reproductive isolation such that RI reaches 1 at low focal plant frequencies with strong positive frequency dependent preference. Shown in Appendix figure C1 is equation C2 evaluated across plant frequencies with $\kappa=0$, and $\rho=0.5$.

**Appendix S3: Using the model with empirical data**

*Phlox example with one pollinator and variation in constancy and preference:*

Pollinator observations of *Battus philenor* were performed on arrays of *Phlox* plants and reported in table S6 by Hopkins and Rausher [1]. These arrays contained 10 light-blue *P. drummondii* plants, 10 dark-red *P. drummondii* plants and 31 *P. cuspidata* plants. Both the number of flower types visited by the pollinators and the number of transitions between each flower type was observed. I calculated RI separately for light-blue and dark-red *P. drummondii* and therefore considered only the light-blue and *P. cuspidata* observations when calculating light-blue *P. drummondii* RI and only dark-red and *P. cuspidata* observations when calculating dark-red *P. drummondii* RI. Below I walk through how to use the observed data to calculate the model parameters for the light-blue *P. drummondii*. Appendix S4 worksheet displays the observed pollinator behavior and calculations for model parameter values for each empirical example including the dark-red *P. drummondii*.

- The frequency of focal plant (light-blue *P. drummondii*) is given.

*f =* 10/41=0.244

- The total visits to light-blue *P. drummondii* (163) and *P. cuspidata* (139) are used to calculate ψ (the proportion of visits to focal plant).

ψ = total visits to light-blue *P. drummondii* divided by total visits to both species

= 163/(163+139) = 0.539

- Preference (ρ) is calculated from proportion of visits to focal plant (ψ) and frequency of focal plant (*f*). This is a rearrangement of equation 1.
  $\rho=\frac{f-\psi}{-f-\psi+2f\psi}$ = 0.568
- Proportion of heterospecific matings (H) observed is calculated from observed pollinator transitions to light-blue *P. drummondii* from light-blue *P. drummondii* (73) and to light-blue *P. drummondii* from *P. cuspidata* (87).

Observed H = 73/(73+87) = 0.54

- Constancy (*k*) is calculated using equation 2.

$\kappa=\frac{\left( 1-H \right)-\psi}{\left( 1-H \right)+\psi-2\psi(1-H)}$ = -0.165

With preference (ρ) and constancy ($\kappa$), the model can be used to predict RI for any frequency of light-blue *P. drummondii* and *P. cuspidata*.

*Ipomopsis example with two pollinators and two locations*:

Pollinator visitation to an array of *Ipomopsis aggregata* and *I. tenuituba* were reported by Aldridge and Campbell in Table 1 of their paper [2]. Two dominant types of pollinators were observed – a hawkmoth (*Hyles lineata*) and hummingbirds (*Selasphorus platycercus*, *S. rufus*, and *Archilocus alexandri*). The arrays contained 8 plants per species and transitions within and between species were reported. The relative frequency of visits by each pollinator into the arrays ($\phi$) was determined by dividing the total visits by the focal pollinator to both *Ipomopsis* by the total visits observed by both pollinators to both species. For example, at Grizzly Ridge hummingbirds visited 145 *I. aggregata* and 14 *I. tenuituba* for a total of 159 plants and hawkmoths visited 65 *I. aggregata* and 248 *I. tenuituba* for a total of 313 plants. The relative frequency of hummingbird visits is 159/(149+313) = 0.3. All other parameters are calculated in Appendix S4 worksheet based on the observed pollinator transitions within and between plants.

**Appendix References**

[1] Hopkins, R. & Rausher, M.D. 2012 Pollinator-mediated selection on flower color allele drives reinforcement. *Science* **335**, 1090-1092.

[2] Aldridge, G. & Campbell, D.R. 2007 Variation in pollinator preference between two Ipomopsis contact sites that differ in hybridization rate. *Evolution* **61**, 99-110. (doi:10.1111/j.1558-5646.2007.00008.x).
